# Relationship between Behavior and Periodontal Health Self-Perception in Diabetic and Non-Diabetic Patients from Transylvania, Romania—A Self-Report Study, including The Desire to Use a Mobile App for Oral Care Improvements

**DOI:** 10.3390/medicina59081419

**Published:** 2023-08-03

**Authors:** Ariadna Georgiana Badea (Paun), Vlad Ioan Bocanet, Iulia Clara Badea, Radu Chifor, Livia Terezia Duma, Cristina Maria Borzan

**Affiliations:** 1 Department of Public Health and Management, Iuliu Hațieganu University of Medicine and Pharmacy, 400012 Cluj-Napoca, Romania; badea.ariadna@umfcluj.ro (A.G.B.); maria.borzan@umfcluj.ro (C.M.B.); 2 Department of Manufacturing Engineering, Technical University of Cluj-Napoca, 400114 Cluj-Napoca, Romania; vlad.bocanet@tcm.utcluj.ro; 3 Department of Preventive Dental Medicine, Iuliu Hatieganu University of Medicine and Pharmacy, 400083 Cluj-Napoca, Romania; iulia.badea@umfcluj.ro; 4 Center for Diabetes, Nutrition and Metabolic Diseases, Cluj County Emergency Clinical Hospital, 400006 Cluj-Napoca, Romania; livduma@yahoo.com

**Keywords:** self-report and monitoring of periodontal signs, oral health, gingival bleeding, tooth mobility, mobile app, oral health screening and monitoring

## Abstract

The study aimed to assess self-reported symptoms of periodontal disease (gingival bleeding, tooth mobility and halitosis) among diabetic and non-diabetic patients from Transylvania, Romania. Using statistical analysis methods, correlation between the aforementioned symptoms and diet, oral hygiene habits, stress, physical activity, and BMI (body mass index) were researched. Another secondary objective was to assess the impact of self-reported oral health, aesthetics, and halitosis on their life. Patients’ willingness to use a mobile app for generating awareness of oro-dental complications, improving their knowledge of oro-dental health, and reminding them to visit the dentist based on an awareness-raising function, was also assessed. *Methods:* The study was based on an original self-administered questionnaire, applied to 182 subjects, in an unselected, randomized manner. A total of 110 questionnaires were applied in two dental offices and 72 questionnaires were applied in a public clinic for diabetic patients from Cluj-Napoca, Romania. *Results:* Warning signs and symptoms relevant to periodontal disease were identified by respondents, having statistically significant associations with the declared oral health-care habits, including interdental aids, vicious habits (nail biting, bruxism), etc. Some of these periodontal signs could be correlated with a certain lifestyle, such as the perceived stress, smoking status, practicing maintenance sports, and alcohol consumption. A total of 66% of the non-diabetics and 68% of the diabetics of the surveyed subjects consider a software application for generating awareness of oro-dental complications useful, and are willing to pay around EUR 6 for it. *Conclusions:* Having a medical condition such as diabetes makes patients pay more attention to dental health compared with non-diabetic patients. Patients expressed their desire to use a software application to help them to be aware of their condition and for improving their self-report capabilities, including their oral-health-related status.

## 1. Introduction

Untreated oral conditions, including dental caries, severe periodontitis, and edentulism affect about 3.5 billion people worldwide, being highly prevalent in the world [1,2,3]. Systemic medical conditions can have a negative impact on oral health [4]. Many adults with diabetes have poor awareness of oral care and health complications associated with diabetes and are receiving limited advice from healthcare professionals [5]. Due to lack of information related to the importance of good oral health in patients with diabetes, doctors need training to offer proper advice. Patients’ quality of life is affected by a variety of factors such as age, level of education, frequency of brushing, duration of diabetes, and referral to a dentist by a physician [6]. Lack of knowledge about the oral complications of diabetic patients leads to the need for education programs supported by well-trained dental and medical professionals [7].

Halitosis or oral malodor commonly known as “bad breath” often leads to anxiety and psychosocial embarrassment. Halitosis has a prevalence ranging from 50% in the USA to 23% in China [8]. There is a positive relationship between HbA1c levels and halitosis among diabetic patients [9] and the level of gingival inflammation.

With more than five billion mobile phone users in 2019 [10] this technology has a great potential to be used to educate the patients in the self-diagnosis and self-monitoring of diseases to improve their oral health status. A study performed in 2018 found that the quality of the 33 software applications evaluated was generally poor, after examining their content and ability to promote oral health [11]. Given the current level of use and accessibility of mobile technology and the lack of information on oral health, this could be an opportunity in developing mobile applications to address this public health challenge.

Nowadays we are witnessing an increase in the incidence and prevalence of chronic non-communicable diseases associated with multi-morbidities. These chronic conditions include chronic periodontal disease and dental caries. It is well known that both diseases have bacterial plaque as the main etiological factor, and primary, secondary, and tertiary prevention is conditioned by the compliance of the subjects, among which are maintaining optimal oral hygiene and regular dental check-ups, and the active involvement of the subjects in the prevention of these diseases.

Eke et al. tried to find an alternative, valid, and reliable—but less resource-demanding—approach for the sustained surveillance of periodontitis at all levels, especially among public health programs with limited resources. Their study demonstrates the potential performance of self-reported measures for use in public health surveillance of periodontitis in the US adult population and for population-based research [12]. Relevant questions about periodontal status were conceived regarding oral hygiene habits, medical history of gum disease, tooth appearance and tooth loss, which were correlated with demo-graphic/risk factors through statistical analysis by calculating specificity, sensitivity, and receiver-operating characteristic statistics [13,14,15].

According to the findings in the literature, an original questionnaire was conceived to point out self-perceived relevant warning signs and symptoms of periodontal disease, and also self-reported local, general risk factors and health conditions that may interfere with periodontal disease.

Our team conducted a cross-sectional, questionnaire-based survey on diabetic and non-diabetic patients from a limited geographical region, Central Transylvania, Romania. The study aimed to assess self-reported symptoms of periodontal disease (gingival bleeding, tooth mobility and halitosis) among diabetic and non-diabetic patients from Transylvania, Romania. Using statistical analysis methods, the correlation between the aforementioned symptoms and diet, oral hygiene habits, stress, physical activity, and BMI (body mass index) were searched. Another secondary objective was to assess the impact of self-reported oral health, aesthetics, and halitosis on their life. Patients’ willingness to use a mobile app for generating awareness of oro-dental complications, improving their knowledge of oro-dental health, and reminding them to visit the dentist based on an awareness-raising function was also assessed.

## 2. Materials and Methods

A cross-sectional, questionnaire-based study was carried out between 28 February 2020 and 28 March 2020. To achieve the objectives, we administered in an unselected, randomized manner an original self-reported questionnaire to diabetic and non-diabetic patients in dental and medical institutions in Cluj-Napoca, Romania. The questionnaire was distributed among patients from 1 public clinic for diabetes (the most important one in North-West Romania) and 2 private dental offices from Cluj-Napoca, Romania, which agreed to offer the questionnaire to their patients. Ethical committee approval was obtained; no. 25/27 January 2020 of Iuliu Hațieganu University of Medicine and Pharmacy, Cluj-Napoca, Romania. All participants in the study signed the informed consent when they completed the questionnaire. An original and anonymous questionnaire was designed that was completed by the patients who contacted the 2 dental offices for various dental conditions. The same questionnaire was completed by the diabetic patients attending a public medical clinic for diabetic and metabolic disorders. The questionnaire consists of 61 questions, including the personal and social data of the respondent, educational level, and profession (occupation) (Appendix A). Gingival inflammation and periodontal status were evaluated using self-assessment related questions about gingival bleeding, tooth mobility, family medical history, gingival recessions, and the presence of bacterial plaque. Other questions were related to dental hygiene habits including number of brushings, types, and frequency of use for auxiliary oral hygiene means, professional dental treatment history, number of meals per day, favorite foods, stress, aesthetic self-perception of the teeth, halitosis self-assessment, weight and height for the BMI calculation, alcohol consumption, physical activity, fixed or removable prosthesis, and the impact of oral health and halitosis on their life. The last three questions were related to the need for and utility of a mobile app for self-assessment and monitoring of the oral diseases and periodontal disease especially, generating awareness of oro-dental complications and improving their knowledge of oro-dental health. The desire to use such a mobile app will be measured by the amount of money that the respondents are willing to pay for it. The variables, and their measurement level and scale are presented in Appendix B.

- The questionnaire
  The questionnaire was validated on a small sample of patients, to assess the understanding and clarity of the wording. The feedback was used to improve the clarity of the items in the questionnaire. The internal consistency of the questionnaire was assessed using Chonbach’s alpha. Because different parts of the questionnaire focused on different aspects, the questions to be analyzed were grouped by subject.

- Inclusion and exclusion criteria
  Included in the criteria of the patients was the commitment of the diabetics and non-diabetic patients who accepted, to completing the questionnaire. The criteria for including patients in the study were: over 18 years old, understands spoken and written Romanian, coherent and conscious, in full mental faculties, acceptance of participation in the study and completion of the questionnaire, presentation at one of the two dental offices or the public clinic for diabetes during the study period (20 February–28 March 2020), and of urban or rural origin. There was no discrimination of gender, religion, education level, or ethnicity. The patient could be non-diabetic, or of type 1 or 2 diabetes. The exclusion criteria were age under 18 years, refusal to participate in the study, dental medical-surgical emergencies, hypoglycemic or hyperglycemic coma, uncooperative, or unconscious patient.

- Data correlations and tested hypotheses
  In the current study, an assessment was made of the association of the main clinical signs of periodontitis with the items in the questionnaire:

1. We tested whether there were associations between periodontal disease symptoms and lifestyle for both types of respondent (with and without diabetes) and for all respondents together.
2. The association between bleeding gums (Q7_1), tooth mobility (Q17), gingival bleeding frequency (Q21), and bad breath (Q41) with:

- Frequency of visits to the dentist (Q14);
- Presence of tooth extractions (Q23);
- Performing orthodontic treatments (Q24);
- Use of dental flossing (Q25), frequency of use (Q27)
- Use of prostheses (Q53);
- Presence of vicious habits (nail biting, bruxism) (Q56);
- Existence of gingival treatments (Q31);
- Number of teeth present (Q39);
- Existence of problems in the oral cavity in the last 6 months (Q43).

3. We checked for significant association between gingival bleeding (Q7_1), tooth mobility (Q17), bleeding frequency (Q21) and bad breath (Q41) and the following factors:

- Gum disease of family members (Q19);
- Age at which the respondent was diagnosed (Q20);
- Aspects of nutrition: belonging to a food group (Q32), the most common place to eat (Q33), following the main meals of the day (Q34), the composition of the daily menu (Q35), the amount of water consumed in a day (Q36), table fat content (Q37);
- Sports activity carried out (Q50).

Hypotheses that were tested for diabetic patients’ answers compared with non-diabetic patients: there is no association between the existence of gingival bleeding and never flossing. There is no association between the existence of gingival bleeding and frequency of flossing. There is no association between the existence of gingival bleeding and the use of mouthwash. There is no association between the existence of tooth mobility and the existence of vicious habits (nail biting, bruxism). There is no association between the existence of diabetes and the self-assessment of bad breath. There is no association between the existence of diabetes and the perception of bad breath by others.

The patients completed the questionnaire before receiving the indications for maintaining good general and oral health. They benefited from all the necessary treatments according to the diagnosis and the treatment plan, regardless of their desire to participate in the study or the answers they filled in in the questionnaire. The χ^2^ test was used to test the hypotheses to determine if there was an association between the variables studied. The variables were nominal, and the independence of the observations was ensured by the way the questionnaire was distributed.

- Statistical analysis

A binary logistic regression analysis was performed to determine the significant factors that predict tooth mobility. Three categories of factors were considered: local predisposition factors (presence of dental plaque (Q15) and dental restorations (Q53)), general risk factors (periodontal disease in the family (Q19), smoking (Q8), drug treatments (Q13), stress (Q38), type of nutrition (Q32), age, sex, presence of physical activity (Q50)) and systemic factors (the presence of diabetes, general diseases (Q10) and obesity). Information about the weight and height of each person was coded into the body mass index (BMI). The BMI was calculated by dividing the weight in kg by the square of the height in m2 [1]. Using the BMI and the WHO guidelines [1] as outlined in Table 1, the respondents were split into two categories: not obese (BMI < 30) and obese (BMI > 30).

The assumptions necessary for this analysis were tested. The dependent variable (tooth mobility) is dichotomous, and was coded as 0 for no mobility present and 1 for tooth mobility present. The independent variables were either dichotomous (Q15, Q53, Q19, Q8, Q13, Q38, Q50, diabetes, Q10, obesity) with 0 for “no” and 1 for “yes” and 1 for “female” and 2 for “men” in the case of sex, or with multiple categories (age—18–26, 27–41, 42–56, 57–75, >75 years old, type of nutrition—vegetarian, lacto-vegetarian, mixed, raw-vegan). Independence of observations was ensured through the collection process of the responses. As there are no continuous variables, the condition of a linear relationship between independent variables and the log odds did not need to be tested. Multicollinearity was also tested by using Spearman’s correlation. Any significant correlations did not cause any issues, all of them being under the value of 0.7. The cutoff value for the dependent variable was set at 0.5. The last category was chosen as the reference for each variable.

## 3. Results

A total of 182 respondents participated in the study, among them 72 diabetic (4 with diabetes type 1 and 68 with type 2) and 110 non-diabetic adults. The acceptance rate for completing the questionnaire was 96%.

**Figure 1:**
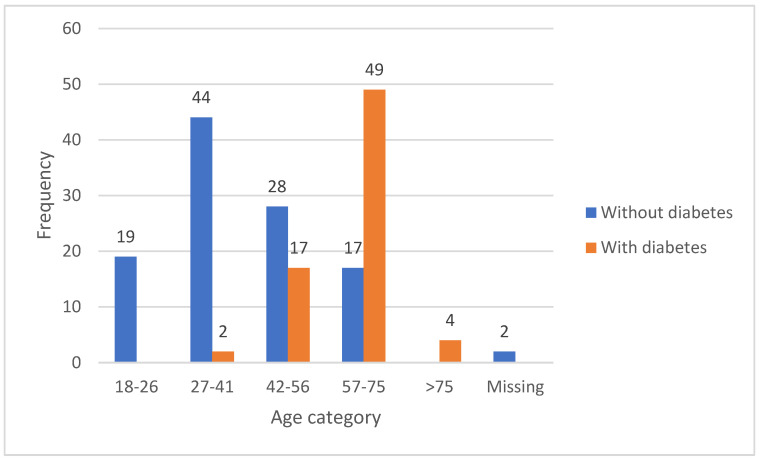
Age distribution of respondents: With diabetes (>75 years: 4; 57–75: 49; 42–56 cured diabetes: 17; 27–41 cured diabetes: 2) and without diabetes (>age 75:0; 57–75: 17; 42–56: 28; 27–41: 21).

Only 181 of the respondents declared their gender, with 63.18% of them being female and 36.81% male (Figure 1). No minors were involved in this study, and only patients over the age of 18 were included.

Most respondents were women from both the non-diabetic group (67%) and the diabetic group (57%) (Figure 1). Most respondents came from urban areas (81% without diabetes, 78% with diabetes). In terms of education, the majority of respondents without diabetes had a university education (64%) while the majority of respondents with diabetes had a high school education (55%) and only 23% had a university education (Table 2).

Table 3 shows the reliability measures for each analyzed subject. All results are in an acceptable range except for the items relating to dental hygiene habits. This can be explained by the very diverse aspects of hygiene habits and treatment history.

### 3.1. Evaluating the Association between the Self-Reported Periodontal Symptoms (Gingival Bleeding, Tooth Mobility, Halitosis) and the Lifestyle, Declared Oral Hygiene and Risk Factors for Periodontal Disease for All Respondents

In Table 4 we present the results which are statistically significant, obtained in the evaluation of the associations of the items in the questionnaire. The measured association average power is represented in the last column of the table.

Gingival bleeding during tooth brushing, flossing, mastication or spontaneous gingival bleeding: 12.5% diabetics reported no gingival bleeding, 45.37% non-diabetics reported no gingival bleeding.

In this study the periodontal clinical examination of the respondents was not carried out, the questionnaire being anonymous.

### 3.2. Binary Logistic Regression Analysis

A few models were tested, the first one using all the mentioned variables (full model) through the backward stepwise (conditional) method, meaning that the removal of a variable is carried out “based on the probability of the likelihood-ratio statistic based on conditional parameter estimates” [17]. The resulting models are presented in Table 5.

The predicted (dependent) variable is tooth mobility, and the considered predictor (independent) variables are: presence of dental plaque, presence of dental restorations, periodontal disease in the family, smoking, drug treatments, stress, age, sex, presence of physical activity, the presence of diabetes, the presence of cardiovascular disease, and obesity.

Age and the presence of diabetes are significant predictors in all the tested models. According to the recommendations of BRFSS, sensitivity and specificity values of >80% are considered to have high validity [13]. Model 9 shows the best performance from a specificity and sensitivity point of view, and is the preferred model. The model explained 68.7% of the variance (Nagelkerke R Square). The model equation is presented in Equation (1) [16].

(1) $$\begin{array}{ll} tooth\;mobility= & -1.093+0.512*peridontal\;disease_{family}-0.702*stress\\ & -20.509*age_{18-26}-1.563*age_{27-41}-1.745*age_{42-56}+0.808\\ & *age_{57-75}-1.058*physical\;activity+2.654*diabetes \end{array}$$

The characteristics of the chosen model are briefly presented in Table 6.

The odds of having tooth mobility increases by 14.213 times when diabetes is present compared to when it is not present, all other factors remaining constant. The odds of tooth mobility vary depending on the age category the respondent is in. Age resulted in being a significant predictor as a whole (*p* = 0.001), but none of the age categories were significant, so it should be interpreted with caution. As age increases, the risk of tooth mobility also increases.

### 3.3. The Desire to use a Mobile App for Generating Awareness of Oro-Dental Complications and Improving their Knowledge of Oro-Dental Health

Diabetic and non-diabetic patients have a similar perception of the usefulness of a software application that maximizes awareness of oro-dental complications and improves their knowledge of oro-dental health (68% vs. 66%). Regarding the importance given to such a software application, quantified by the maximum amount they are willing to pay, there is a significant difference between the group of patients with diabetes and those without diabetes. A total of 97% of those with diabetes are willing to pay over 6 EURO, most of them being over EUR 8. Only 31% of patients who do not have diabetes choose to pay more than RON 30 for such a software application installed on the phone, with most (69%) being willing to pay less than EUR 6.

## 4. Discussion

The purpose of this study was achieved; data were collected from 182 questionnaires completed by diabetic and non-diabetic patients. Statistical processing of the self-report questionnaire suggests that certain signs of periodontal disease (gingival bleeding and tooth mobility) had predictable associations with the declared use of auxiliary hygiene aids (dental floss or interdental toothbrush), vicious habits (nail biting, bruxism) or halitosis. The desire of the patients to use a mobile app for generating awareness of oro-dental complications, improving their knowledge of oro-dental health, and reminding them to visit the dentist was measured by the amount of money that respondents declared they were willing to pay.

Comparing the results of our study with the ones mentioned in the literature, some similarities but also differences were identified.

Baudet et al. presented in their study in 2020 the fact that among 794 adults interviewed, 63.2% reported gingival bleeding (GB) [18]. In our group of respondents, 54.62% of non-diabetics and 87.5% of the diabetics said they had spontaneous GB either during tooth brushing and/or mastication.

Bowyer et al. performed a study in 2011 in which 229 questionnaires were completed in 14 general medical practices. Respondents were typically older adults, who stated that they were from a White British ethnic background having type 1 (7.2%) or type 2 (87.0%) diabetes, with other/unsure 5.8%. Most respondents (79.8%) visited a dentist at least once or twice a year, but oral care habits varied, so 67.2% reported brushing at least twice a day, whereas only 15.3% flossed daily [5]. In our study the use of the dental floss or other interdental aids (interdental brush) have been reported for 46.22% from the non-diabetic group and 19.44% for the diabetics. Respondents who never used any interdental aids (dental floss or interdental brush) accounted for 15.9% for the non-diabetic group and 52.75% of the diabetics. A total of 52.72% of the non-diabetic patients reported regular dentist check-ups 1 or 2 times per year, and 45% visited a dental office occasionally. For diabetic patients, a regular check-up 1 or 2 times per year was reported by 65.27%, and 34.72% visited a dental office occasionally. Bowyer et al. reported that 43% of the patients were very aware of mouth dryness being associated with diabetes complications. A total of 13.1% reported swollen or tender gums, and 12.8% loose teeth. In our study, 26.6% of respondents without diabetes and 97.14% with diabetes reported having a dry mouth. Sadeghi et al. [6] performed an analytical cross-sectional, self-reported questionnaire study taking into consideration 200 diabetic patients, consisting of 88 men and 112 women with a mean age of 55.2 years; 36.5% were 50 years old and less and 63.5% were over 50 years old. Their educational level was non-academic 70.5% vs. academic 29.5%. Regarding the level of education, in our group 23% of the diabetic patients and 64% of non-diabetic patients had university studies. Compared to the Sadeghi et al. group, where 50.5% were smokers and 49.5% non-smokers, in our group the non-smokers were 82.40% non-diabetic patients and 75% were in the diabetic group. Also, in our study, those who smoked at least one packet of cigarettes a day represent 4.62% of the non-diabetic group and almost 21% of those with diabetes. Considering the etiology of tooth extraction, in Sadeghi’s study, for the ones who reported an etiology of tooth extractions, mobility was 2%, caries 27%, mobility and caries 44.5%, and no missing teeth were reported by 26.5%. In our study, 56% of the non-diabetic patients reported that extractions were due to dental decay, and 66.66% of the diabetics reported dental decay and periodontal disease as the main causes of extractions, with 29.16% of them reporting dental decay as the main cause of extractions.

In Sadeghi’s study, 50.5% reported that their frequency of brushing was at least once a day; no brushing was reported by 49.5%, once a day by 43.5% and twice a day by 7%. For frequency of dental visits, 83% reported regular checkups [6], which were higher percentages when comparing their results with ours: 52.72% for non-diabetics vs. 65.27% for diabetics.

Two hundred questionnaires and consent forms were distributed to the diabetic patients in Dubai, United Arab Emirates, in Rashid Hospital [7]. The mean participant age was 47 years and 36% were smokers, more than the patients from our study (17.6% smokers who were non-diabetic and 25% of diabetics), and fewer than those of Sadeghi et al. (50.5% smokers). In Eldarrad’s study, 87% of diabetic patients were dentulous and 13% were edentulous; fewer edentulous that the percentage of diabetic patients in our study (23.61%). Of the edentulous participants, 34% were wearing complete dentures, which was more than in our study, where 23% were wearing complete dentures and 19.44% natural teeth and removable dentures.

In the study by Eldarrad [7], 77% of participants were suffering from a dry mouth. In our study, 26.60% of non-diabetics and 97.14% of diabetics reported having a dry mouth. Most of the participants (70%) in Elderrad’s self-reported questionnaire study were aware that bleeding during brushing is a sign of gingival disease. In our study, 54.62% of non-diabetic participants and 87.5% of diabetics reported gingival bleeding, both spontaneous and/or during brushing, flossing, and mastication. 

In Elderrad et al.’s study, 19% of the respondents did not use a brush daily, 31% brushed twice a day, and a significantly higher number of respondents (50%) brushed once a day; these are higher percentages compared with our results, in which 40.36% of non-diabetics and 26.76% of diabetics brush their teeth daily.

In their study, 66% never used dental floss, 11% reported using dental floss once a day, and 23% did not use it daily. Dental floss was used less, compared to our results: 50% never, 19.44% used daily and 30.56% occasionally. A total of 40% reported yearly visits to a dental clinic and 14% reported they visited for a regular dental check-up, a lower percentage compared with our study [7].

Another study showed that from 38 type 2 diabetic patients, sixteen subjects (42.1%) reported halitosis, significantly correlating with higher HbA1c levels [9]. In our study, 54.16% of diabetics reported self-perceived halitosis or halitosis by others, and 24.52 of non-diabetics reported self-perceived halitosis. Respondents with diabetes reported significantly more often that others perceived them as having bad breath than respondents without diabetes (Table 4).

Sandberg et al. included in their study 102 randomly sampled diabetic patients, from which 53.5% reported halitosis, 62% natural teeth, 25.5% natural teeth and dentures. In their study, 102 age- and gender-matched non-diabetic subjects were also included, and among these 28.4% reported xerostomia (dry mouth), 70% had natural teeth and 21.6% wore combinations of natural teeth and dentures. A toral of 12.74% subjects in the diabetic and 6.86% in the control group were totally edentulous [19].

In our study, the percentage of dry mouth in non-diabetic patients (26.6%) is similar to the data from the literature [19], but for the diabetics almost all respondents (97.14%) reported xerostomia (dry mouth), while in the literature we found values for the diabetic patients of between 43% and 77%. High values of xerostomia in our patients with diabetes could be explained by the mean age (62.43), education level (23% with university studies, 55% with high school education), HbA glycated values (49.57% had optimal glycemic control with HbA glycated <7; 27.73% had poor glycemic control with HbA glycated >8), the number of edentulous patients (38.88% of the diabetic patients had no teeth or very few), and 65.27% had regular visits to the dental office. There was a prevalence of self-rated oral health (halitosis, dry mouth, tooth mobility, and gingival bleeding). A total of 19.44% of the diabetic group use dental floss or an interdental brush daily, and 57.46% never use dental floss or an interdental brush.

None of the diabetic respondents use an air flosser or water irrigator, while 34.72% use mouth water on a daily basis and 50% never use mouthwash.

Another study from Brazil analyzed socioeconomic variables, gender, heredity, capillary glucose control and local factors (prosthesis, dry mouth sensation) in 196 diabetic and non-diabetic patients. This study reported that changes in the oral cavities can be generated by the use of prosthetic appliances and not so much by the presence and the absence of type 2 diabetes [20]. In our study regarding wearing removable prostheses, 9.43% of non-diabetic patients reported that they have natural teeth and removable prosthesis, with only one person having removable full dentures vs. 19.44% of diabetics reporting having natural teeth and a removable prosthesis, and 23.61% of them having removable full dentures. Verhulst et al. enrolled 764 patients with type 2 diabetes in their study from 24 offices in the Amsterdam area in the Netherlands [21]. A total of 16% of them were smokers, a lower percentage compared to our patients, and 25% were diabetic. In their study, about a quarter (24%) reported not visiting a dentist regularly, and 30% did not have dental insurance coverage; in ours, 52.72% of non-diabetics and 65.27% of diabetics reported regular check-ups, at least annually. A total of 16% of their patients were edentulous and had full dental prostheses, while 29% had a partial dental prosthesis. In our study, 23.61% of diabetic patients are edentulous and 19.44% have a partial removable prosthesis. Pain in the mouth, dry mouth and bad breath were reported by 15%, 37% and 12% of the patients, respectively, in their study. In our study 26.6% of non-diabetics and 97.14% of diabetics reported dry mouth. In Verhulst et al.’s study, almost 70% suffered from periodontitis [21], while in our study 23.94% of diabetic patients were diagnosed after 40 years old with periodontal disease and 35.21% were diagnosed after 50 years old.

Grover et al. showed in their study that there is an association of sleep deprivation with the severity of periodontal disease [22], and Pereira et al. concluded that individuals with self-reported sleep disorders presented with worse self-perceived oral health for most variables [23]. Our findings sustain their conclusion because significantly fewer respondents who have difficulty sleeping or relaxing report having gingival bleeding (Table 4).

In another study, 567 people from marginalized populations in two Canadian provinces were included to respond a questionnaire about self-rated oral health and experiences of accessing and receiving healthcare, standard self-report measures of health and quality of life, and socio-demographic information. The prevalence of self-rated poor oral health was high, with almost half (46.3%) of the participants reporting poor or fair oral health [24]. In our study, the percentage is even higher; 93.05% of diabetics and 40.74% of non-diabetics answered that dental problems affected their quality of life. Wallace et al.’s study found that significant relationships were observed between poor oral health and vulnerabilities related to mental health: depression (52.5% vs. 7.6% reporting a mood disorder), arthritis (38.6% vs. 15.9%), high blood pressure (31.8% vs. 17.7%), and diabetes (13.2% vs. 6.6%). Almost half of the participants (46.4%) reported poor oral health [24]. In our study, 12.96% of non-diabetics and 6.94% of diabetics had lost their interest or pleasure in ordinary acts in the last month, and 24.77% of non-diabetics and 6.94% had felt sad or demoralized in the last month.

In a representative sample of the Portuguese population, 2017 of 1102 individuals answered a questionnaire reporting that 97.6% brushed their teeth daily [25], a much higher percentage than the ones from our study or Elderrad et al.’s. In Melo et al.’s study, 70.3% had lost permanent teeth and 6.4% were edentulous, while in our study 22.18% of non-diabetics and 0% of diabetics had no extractions of permanent teeth. In their study, 50.1% of the participants had experienced difficulty eating and/or drinking, 18% had felt ashamed of the appearance of their teeth, and 69.3% had experienced toothache or gingival pain [25]. In our study, 3.84% of non-diabetics and 27.77% of diabetics reported difficulties in chewing and eating, and 3.8% of non-diabetics and 43.05% reported difficulties in phonation and speech in the last 6 months.

In our study, 44.44% of the non-diabetic respondents were satisfied with their teeth appearance vs. 72.83% of the diabetics group, despite the fact that there were a great number of extractions in the diabetic group. An explanation could be the mean age of the respondents of the diabetic group, where the standards of dental aesthetics are no longer at the forefront, with the general state of health prevailing.

In our study, another evaluation that has a paradoxical association, and one that we would not have expected, is the association of those who have benefited from gingival treatments with the ones that report tooth mobility (Table 4). There was a significant association, according to statistical analysis (average power (Cohen 1988) Cramer’s V).

- A possible explanation would be that it is especially the respondents with perceived tooth mobility who ask for gingival treatments.
- Another explanation is that these gingival treatments take place occasionally.
- Or, that these gingival treatments are not performed by well-trained periodontists. The periodontology specialty has been introduced in Romania recently. There are also no dental hygienists or prophylaxis assistants in Romania. Only a few generations of hygienists had been trained many years ago.
- Another explanation would be the fact that dental treatments are not followed by optimal oral hygiene, including interdental space hygiene, which is also demonstrated in this study (by the high percentage of those who do not use interdental hygiene aids either at all or occasionally).

The results of our study regarding the existence of gingival disorders in the family agree with the data from the literature, which show a family predisposition in patients with periodontal disease among non-diabetic patients (Table 4). There was a significant association of the self-reported periodontal symptoms (bleeding, tooth mobility, and oral malodor) with oral hygiene habits and lifestyle patterns (Table 4), in accordance with the data from the literature, which compare the oral hygiene status, habits and lifestyle patterns with dental and periodontal parameters. Considering these results, we can state that the respondents in our study are in the phase of self-reported periodontal signs (gingival bleeding, oral malodor, and tooth mobility).

Data related to the determinants of oral health, and genetic, biological and environmental, but also behavioral and social factors, lifestyle, and well-being habits, can be collected using information and communication technology. This will help us in building prevention strategies, and not only treating and integrating oral health into general health [26]. Previous studies show low awareness and knowledge of diabetes-related complications, compared with awareness and knowledge of the systemic complications of diabetes [1,7]. Regarding oral health, there is a lack of information among patients, especially for the ones that needed more oral health, the diabetics. An important part of our study was dedicated to finding out the difference between the perceived usefulness of having a software application to help patients to become aware of oral cavity problems in diabetics and non-diabetics. This software application should help the patients improve their self-report capability for oral health and monitoring periodontal disease, having an awareness-raising function, and reminding them to visit the dentist. The study intended to evaluate the desire to use a mobile app. Diabetic and non-diabetic patients have a similar perception of the usefulness of a software application that maximizes awareness of oro-dental complications and improves their knowledge of oro-dental health: 68% vs. 66%. Regarding the importance given to such a software application, quantified by the maximum amount they are willing to pay for it, there is a significant difference between the group of patients with diabetes and those without diabetes. A total of 97% of those with diabetes are willing to pay over EUR 6, with most of them being willing to pay over EUR 8. Only 31% of the patients who do not have diabetes choose to pay more than EUR 6 euros for such a software application installed on the phone; most of them, 69%, are willing to pay less than EUR 6.

The large number, 47 of the diabetic and non-diabetic patients, who would find such a software application useless, out of a total of 181 respondents, may be due to the absolute novelty of using such a software application, not having had such an opportunity before the digital era. However, only 19 diabetic and non-diabetic patients—10.5% of those who answered the questionnaire—consider such a software application completely useless, most of them non-diabetic patients.

On the other hand, these results encourage us to believe that the transition from the stage of self-report to self-care oral health, could take place in an accelerated way and impact a larger number of subjects using a mobile app, considering the availability of the app and the consistent percentage of the respondents who would pay an amount of money for this application.

The state-of-the art use of eHealth technologies—and of mobile eHealth technology in particular—will have fostered a more collaborative approach to oral healthcare, as well as an improved access to expert knowledge for all, in urban and remote areas, in developed and developing countries [26].

The novelty of this study is especially related to the evaluation of patients’ desire to use a mobile application that will help them improve their oral health. As far as we know, there have been no such studies in Transylvania, Romania. The symptoms of periodontal damage (gingival bleeding, tooth mobility and halitosis) also correlated more with oral hygiene, diet, and bad habits for non-diabetic patients, compared to diabetic patients.

Our study has certain limitations: the small number of patients included and the limited number of dental offices where the questionnaire has been distributed. Further investigations are needed with the help of a minimum viable product of this type of software application tested on a larger group of non-diabetic and diabetic patients. The collected feedback from them will be helpful for a better understanding of their needs to improve self-diagnosis of oral health status capabilities and their condition in general.

## 5. Conclusions

Even though there was no clinical oral examination, we can conclude, based on the significant associations found in our study, which are in accordance with similar associations in the literature concerning some of these periodontal signs (gingival bleeding, tooth mobility and halitosis), that the respondents from this geographical area are aware of periodontal symptoms.

They also want to improve the self-care process, guided in the future by a mobile app. Most diabetic and non-diabetic patients consider a software application that maximizes awareness of oro-dental complications and improves their knowledge of oro-dental health, useful.

However, diabetic patients are willing to pay a higher amount of money to have access to such a mobile application; probably, due to their general health condition, their concern for oral health increases.

## Figures and Tables

**Table 1:** BMI and nutritional status according to WHO [16].

BMI	Nutritional Status
Below 18.5	Underweight
18.5–24.9	Normal weight
25.0–29.9	Pre-obesity
30.0–34.9	Obesity class I
35.0–39.9	Obesity class II
Above 40	Obesity class III

**Table 2:** The distribution of the level of education by sex for diabetic and non-diabetic patients.

	Category	Non-Diabetic		Diabetic		Total
		Frequency	Percent	Frequency	Percent	
Sex	Men	36	33%	30	42%	36.26%
	Women	73	66%	42	58%	63.18%
	Unreported	1	1%	0	0%	0.54%
	Total	110	100%	72	100%	
Area	Rural	20	18%	16	22%	19.78%
	Urban	90	82%	56	78%	80.21%
	Total	110	100%	72	100%	
Education	Preschool	0	0%	0	0%	0%
	Primary school	1	2%	2	1%	1.65%
	Middle school	8	7%	14	20%	12.09%
	High school	19	17%	38	55%	31.32%
	Post-secondary	6	5%	0	0%	3.29%
	University	70	64%	18	23%	48.36%
	Post-university	4	4%	0	0%	2.19%
	Others	1	1%	0	0%	0.54%
	Unreported	1	1%	0	0%	0.54%
	Total	110	100%	72	100%	

**Table 3:** Internal consistency by subject.

Category (Factors Analyzed)	Question No.	Alpha	N
Gingival inflammation and periodontal status (gingival bleeding, tooth mobility, family medical history, gingival recessions and the presence of bacterial plaque).	7, 10, 11, 12, 13, 15, 16, 17, 18, 19, 20, 21, 22, 23, 24, 30, 31, 39, 43	0.9	36
Dental hygiene habits (number of brushings, types and frequency of use for auxiliary oral hygiene means, professional dental treatment history).	14, 25, 26, 27, 28, 29	0.092	10
Number of meals per day, favorite foods, stress.	32, 33, 34, 36, 37, 38	0.792	10
Aesthetic appearance of the teeth, halitosis self-assessment, weight and height for BMI calculation, alcohol consumption, physical activity, fixed or removable prosthesis.	8, 9, 40, 41, 42, 44, 45, 46, 47, 48, 49, 50, 51, 52, 53, 54, 55, 56, 57, 58	0.728	33
Need for and utility of a mobile app.	59, 60, 61	0.748	3

**Table 4:** The association between the self-reported periodontal symptoms (gingival bleeding, tooth mobility and halitosis) and the lifestyle, declared oral hygiene and risk factors for periodontal disease.

Periodontal Symptom	Lifestyle/Habits	Respondents	χ^2^ Test Association	*p*	Association Average Power Cohen, Cramer’s V
Gingival bleeding	Frequency of flossing (never)	all	17.640	0.001	0.315
	Frequency of flossing (last 7 days)	without diabetes	14.319	0.006	0.361
	Frequency of flossing (last 7 days)	all	23.711	0.001	0.365
	Frequency of mouthwash use (last 7 days)	diabetics	16.382	0.003	0.487
	Speech difficulties	all	27.328	<0.001	0.393
	Masticatory difficulties	all	33.580	<0.001	0.439
	Difficulties in cleaning teeth	all	25.360	<0.001	0.380
	Vicious habits	all	31.396	<0.001	0.421
	Bruxism	all	9.806	0.002	0.232
	Nail biting	without diabetes	9.193	0.002	0.232
	Nail biting	all	20.865	<0.001	0.232
	Existence of sleep disturbances	all	20.298	<0.001	0.339
	Existence of discomfort and restraint during the smile	all	36.260	<0.001	0.451
	Practice of sport	all	12.704	0.001	0.267
	Gingival disorders in the family	all	11.774	0.003	0.259
Tooth mobility	Vicious habits	all	17.224	<0.001	0.314
	Gingival disorders in family	without diabetes	7.040	0.030	0.255
	Gingival disorders in family	diabetics	34.244	<0.001	0.715
	Gingival disorders in family	all	36.666	<0.001	0.458
	Practice of sport	diabetics	9.961	0.002	0.386
	Practice of sport	all	10.976	0.001	0.249
	Existence of gingivitis treatment	all	18.510	<0.001	0.324
	Missing teeth	all	9.164	0.002	0.234
	because of dental caries and tooth mobility	all	4.989	0.026	0.172
Halitosis	self-assessment	diabetes	17.414	<0.001	0.313
	the perception of bad breath by others	diabetes	48.132	<0.001	0.517

**Table 5:** Summary of logistic regression models to predict tooth loss.

Predictor Variables	Model									
	1	2	3	4	5	6	7	8	9	10
Local predisposition factors										
Presence of dental plaque	0	0	-	-	-	-	-	-	-	-
Presence of dental restorations	0	0	0	0	0	-	-	-	-	-
General risk factors										
Periodontal disease in the family	0	0	0	0	0	0	0	0	0	0
Smoking	0	0	0	0	0	0	-	-	-	-
Drug treatments	0	0	0	0	0	0	0	0	-	-
Stress	0	0	0	0	0	0	0	0	0	-
Type of nutrition	0	0	0	0	-	-	-	-	-	-
Age	X	X	X	X	X	X	X	X	X	X
Sex	0	-	-	-	-	-	-	-	-	-
Presence of physical activity										
Systemic factors										
The presence of diabetes	X	X	X	X	X	X	X	X	X	X
The presence of cardiovascular disease	0	0	0	-	-	-	-	-	-	-
Obesity	0	0	0	0	0	0	0	-	-	-
Performance measures										
Specificity (%)	91.3	91.3	91.3	91.3	91.3	91.3	91.3	91.3	90	90
Sensitivity (%)	83.3	81.5	83.3	81.5	81.5	79.6	79.6	79.6	83.3	79.6
Overall percentage (%)	88.1	87.3	88.1	87.3	87.3	86.6	86.6	86.6	87.3	85.8

“X” = significant, “0” = not significant, “-” = removed from the model.

**Table 6:** Model characteristics.

Variables in the Equation	B	S.E.	Wald	df	Sig.	Exp(B)	95% C.I. for EXP(B)	
Periodontal disease in the family	0.512	0.267	3.658	1	0.056	1.668	0.987	2.817
Stress	−0.702	0.508	1.908	1	0.167	0.495	0.183	1.342
Age			18.581	4	0.001			
18–26	−20.509	10,956	0	1	0.999	0	0	.
27–41	−1.563	1.516	1.063	1	0.303	0.21	0.011	4.088
42–56	−1.745	1.322	1.744	1	0.187	0.175	0.013	2.329
57–75	0.808	1.276	0.401	1	0.527	2.244	0.184	27.366
Presence of physical activity	−1.058	0.62	2.917	1	0.088	0.347	0.103	1.169
The presence of diabetes	2.654	0.728	13.279	1	0	14.213	3.41	59.247
Constant	−1.093	1.36	0.645	1	0.422	0.335		

## Data Availability

The datasets used and/or analyzed during the current study are available from the corresponding author on reasonable request.

## Appendix A

U.M.F. Iuliu Hațieganu, Cluj-Napoca

QUESTIONNAIRE

Thank you for agreeing to participate in our study. The purpose of the investigation is to conduct a survey aimed at evaluating the risk factors of periodontal diseases at the population level, as a component of a doctoral scientific research.

Your involvement in the research is very important and useful. All information provided by you will be used strictly for scientific purposes, respecting confidentiality, anonymity and the principles of scientific research ethics.

Accepting participation in the study will not have any repercussions on you and you can withdraw at any time.

Please choose the most appropriate answers that you think characterize you:

**1. Age**: .............

**2. Sex**: F/M...................

**3. Live in:**

- rural area
- urban area

**4. Educational level:**

- without
- Primary school,
- Elementary school,
- High school,
- University,
- other, please mention

**5. Profession:**

**6. Occupation:**

**Have you worked in an environment with exposure to occupational hazards?**

- Yes        No

If **Yes**, what kind of hazards:

- physical (noise, vibrations, ionizing radiation, powders, dust, allergens, others—which……),
- chemical (mercury, cyanides, heavy metals, others, which…….),

Duration of exposure, in years..............................................................

**7. Do you have bleeding gums:**

- Yes        No

If **YES**, what type?

- spontaneous gingival bleeding
- Bleeding gums when brushing teeth
- Bleeding gums when using interdental floss or an interdental brush
- Bleeding gums when chewing
- I don’t have bleeding gums

**8. How many cigarettes do you smoke per day?**

- fewer than 7 cigarettes/day
- 1 pack of cigarettes/day
- 2 or more packs of cigarettes/day
- I do not smoke
- between 7 and 15 cigarettes/day

**9. Have you have smoked in the past?**

- Yes        No

**10. Do you suffer from any general conditions? YES/NO**

If **YES**, which one?

- Diabetes
- Allergies
- Cardiovascular diseases, including hypertension
- Gastric ulcer, Helicobacter Pylori infection
- Kidney diseases
- Rheumatoid arthritis
- Osteoporosis
- Chronic lung diseases
- Depression
- Other diseases.
  Which?......................................................................................................................................................................................................................................................................................................................................................................................................

**11. Do you have normal blood sugar?**

- Yes        No   I do not know

**12. Serum cholesterol values are high:**

- Yes        No   I do not know

**13. Do you take any medications?**

- Yes        No

If the answer is **YES** (affirmative), please list the medications or dietary supplements used.............................................................................................................................................................................................................................................................................................................................

**14. How often do you go to the dental office?**

- Occasionally when I have a dental emergency
- Annual regular checkups
- Regular checkups, twice a year
- Occasionally for scaling

**15. At the dental consultation, were you told that you have bacterial plaque?**

- Yes        No

**16. For better/easier visualization of dental plaque have you used disclosing tablets?**

- Yes        No

**17. Do you have tooth mobility (permanent teeth move)?**

- Yes        No

If the answer is yes (**YES**), please select on a scale from 1 to 5, if score 1 = absent tooth mobility and score 5 = generalized tooth mobility:

1. no tooth mobility
2. average mobility
3. increased mobility
4. mobility localized to a reduced number of teeth
5. generalized mobility (in almost all teeth)

**18. At what age were you diagnosed with tooth mobility?..........................................**

**19. Do members of your family have gum disease (periodontal disease)?**

- Yes        No   I do not know

**20. If you have been diagnosed with periodontal disease (tooth mobility), at what age did it start?**

- Before 20 years old
- between 20 and 30 years old
- After 30 years
- After 40 years
- After 50 years
- I do not have periodontal disease

**21. How often do you have bleeding gums?**

- Daily
- Occasionally
- Several times per day

**22. Do you have gingival recession?**

- Yes        No

**23. If you had tooth extractions, they were due to:**

- Complications of dental caries
  Tooth mobility
- Other causes
- I haven’t had any tooth extractions

**24. Have you had orthodontic treatment (to align the teeth)?**

- Yes        No

**25. What dental hygiene products do you use?**

- Dental floss
- Manual tooth brushing
- Mechanical tooth brushing (electric, sonic, etc.)
- Superfloss
- Interdental brush
- Oral irrigator
- Other products. Which one?.....................................

**26. How much time do you spend on dental hygiene in a day?**

- Less than 3 min
- 3 min
- 5 min
- 10 min
- 20 min
- 25 min

**27. How often do you floss or use interdental brush?**

- Daily
- Weekly
- Occasionally
- Monthly
- Never

**28. How many times in the last 7 days, along with the toothbrush, have you used mouthwash or other oral rinse solutions for the treatment of dental conditions?**

- Daily
- Occasionally
- Once every 2 days
- Weekly
- I have never used mouthwash

**29. How many times in the last 7 days, in addition to the toothbrush, have you used other dental hygiene products (dental floss, mouthwash, etc.)?**

- Daily
- Occasionally
- Once every 2 days
- Weekly
- I have never used other oral hygiene products

**30. Do you think you might have gum problems?**

- Yes        No   I do not know

**31. Have you ever had any treatment for gingivitis, for example scaling, root planning, or a treatment called deep professional cleaning?**

- Yes        No   I do not know

**32. In which category, in terms of nutrition, do you think you fit?**

- Vegetarian (vegan)
- Lacto-vegetarian
- Mixed
- Raw vegan

**33. Eat more often at:**

- Restaurant/at work
- home
- Fast-food restaurant (Mc Donalds, BurgerKing, etc.)

**34. Do you eat the main meals of the day?**

- Yes        No

**35. The daily menu consists mainly of:**

- Precooked
- Meals prepared at home

**36. What is the amount of water used in a day?**

- 1 glass of water (approx. 250 mL)/day:
- 3 glasses of water/day
- 5 glasses of water/day
- 7 glasses of water/day
- between 8 and 11 glasses of water/day

**37. Do you prefer a diet?**

- Low in fat
- Rich in fat

**38. Do you consider yourself stressed?**

- Yes        No   I do not know

If **YES**, what are the main sources of stress?

- Family,
- Work,
- I do not know,
- Other, specify.........

**39. Select the option that most faithfully corresponds to the situation known to you, regarding the presence of teeth in the oral cavity:**

1. I don’t have any extractions, (I have 28–32 teeth)
2. I’m missing a few teeth
3. I’m missing quite a few teeth
4. I have almost no teeth left
5. I don’t have any teeth left

**40. Are you satisfied with the appearance of your teeth?**

1. very satisfied
2. satisfied
3. quite satisfied
4. dissatisfied
5. very dissatisfied

**41. Do you feel like you have bad breath?**

- Yes        No

**42. Have those around you noticed that you have bad breath?**

- Yes        No    I do not know

**43. In the last 6 months, how many times have you had problems in the oral cavity, including the teeth, causing:**

A. chewing difficulties and enjoying food:
  - not affected
  - less than once a month
  - once or twice a month
  - once or twice a week
  - every day
B. in speech, phonation and for clear pronunciation
  - not affected
  - less than once a month
  - once or twice a month
  - once or twice a week
  - every day
C. in teeth cleaning
  - not affected
  - less than once a month
  - once or twice a month
  - once or twice a week
  - every day
D. sleep and relaxation disorders:
  - not affected
  - less than once a month
  - once or twice a month
  - once or twice a week
  - every day
E. discomfort and restraint when smiling and exposing teeth when speaking:
  - not affected
  - less than once a month
  - once or twice a month
  - once or twice a week
  - every day

**44. Please state your weight (in kg) and your height (in meters). This is to calculate your BMI (body mass index).**

weight (in kg).................   height (in meters)..................

**45. How often do you drink an alcoholic beverage?**

- never
- monthly or less frequently
- 2–4 times a month
- 2–4 times a week
- 4 or more times a week

**46. How many standard drinks (BS) do you consume in a typical day when you drink?** A standard drink (BS) contains 12 g of pure alcohol and is equivalent to 1 can of beer of 330 mL, 1 glass of wine of 125 mL or 1 shot of 40 mL.

- one or two
- three or four
- five or six
- seven or nine
- ten or more.

**47. How often do you drink 6 or more BS (standard drinks) on one occasion?**

- never
- monthly or less frequently
- monthly
- weekly
- daily or almost daily

**48. Have you lost interest or pleasure in your usual activities in the past month?**

- Yes        No

**49. Have you felt sad, demoralized or helpless in the past month?**

- Yes        No

**50. Do you do regular physical activity, such as maintenance sports?**

- Yes        No

**51.**

**Have you had in the Last 7 Days, due to Problems with the Teeth, in the Oral Area, Jaw or Implants, ...**	Very Often	Often	Occasionally	Rarely	Never
Difficulty pronouncing certain words?					
Feeling like your sense of taste is affected?					
The impression that your life is less and less satisfying?					
Difficulty relaxing?					
**It seemed to you, based on the problems of the last 7 days at the level of the teeth and the oral cavity:**	Very often	Often	Occasionally	Rarely	Never
That you were more tense?					
That you had to interrupt the meals?					
That you didn’t like eating certain foods?					
That you were more irritated with certain people?					
That you found it difficult to complete the daily tasks in your life?					
That you felt powerless to do something?					
That you felt a little embarrassed, ashamed?					
That your diet was unsatisfactory?					
**Have you had in the last 7 days:**	Very often	Often	Occasionally	Rarely	Never
Pain in the mouth?					
Feeling insecure about your teeth, mouth area, jaw or implants?					

**52. Are you generally satisfied with the health of your teeth?**

1. very satisfied
2. satisfied
3. quite satisfied
4. dissatisfied
5. very dissatisfied

**53. Do you have removable dentures ? (Crowns and fixed dental bridges are not taken into account).**

☐ I don’t have removable prostheses

☐ I have natural teeth plus a removable prosthesis in one or both jaws.

☐ I no longer have any natural teeth and have full dentures on my upper and lower jaw.

**54. Do you feel that dental problems are affecting your quality of life?**

- Yes        No

If **YES**, on a scale of 1 to 5, where 1 = not at all and 5 = very much, please rate to what extent your quality of life is affected:

1. not at all,
2. very little,
3. little,
4. a lot,
5. very much.

**55. You have had or have the feeling of dryness in the oral cavity (mouth):**

- Yes        No

**56. You have had or have repetitive addictive habits (parafunctions), such as:**

- Bruxism (teeth grinding)
- Finger sucking
- Nail biting
- The introduction of inedible objects such as: pens, pencils in the oral cavity or between the arches; using dental floss between the teeth,
- other variants of parafunctions (please list).
- I do not have and have not had repetitive vices (parafunctions)

**57. Would you like to use a mobile software application that will help you self-assess the risk of periodontal disease?**

- Yes        No

**58. Would you like to use a mobile software application that will help you improving the oral hygiene and prevention of periodontal disease, having reminders about regular checkups to the dentist?**

- Yes        No

**59. What would be the minimum amount you would be willing to spend to use this phone app (lei—Romanian currency, 1 Euro = 5 lei, approximately)?**

1. between 10 and 15 lei
2. between 20 and 25 de lei
3. between 30 and 35 de lei
4. between 40 and 45 de lei
5. between 50 and 55 de lei
6. over 60 de lei

**60. On a scale of 1 to 5, where 1 = totally useless and 5 = extremely useful, rate the desirability of a mobile phone app for self-assessing the risk of periodontal disease and having reminding function to visit the dentist:**

1. Totally useless
2. a bit useful
3. useful
4. very useful
5. extremely useful

**61. What would be the maximum amount you would be willing to spend to use this phone app?**

1. between 10 and 15 lei
2. between 20 and 25 de lei
3. between 30 and 35 de lei
4. between 40 and 45 de lei
5. between 50 and 55 de lei
6. over 60 de lei

Thank you for your cooperation and taking the time to complete the questionnaire!

## Appendix B

**Variable**	**Question**	**Measurement Level**	**Scale Values**
Age	Q1_1	Scale	numeric
Sex	Q1_2	Nominal	Male/Female
Location	Q2_1	Nominal	string
Environment	Q2_2	Nominal	Rural/Urban
Education level	Q3	Nominal	1 to 6
Profession	Q4	Nominal	string
Current occupation	Q5	Nominal	string
Exposure to environmental factors	Q6_1	Nominal	Yes/No
Exposure to environmental factors (physical/chemical)	Q6_2	Nominal	0 to 2
Exposure to environmental factors (duration in years)	Q6_3	Scale	numeric
Gum bleeding	Q7_1	Nominal	Yes/No
Gum bleeding type	Q7_2	Nominal	string
Smoking—cigarettes per day	Q8	Ordinal	numeric
Did you smoke in the past	Q9	Nominal	Yes/No
General health issues—diabetes	Q10_1	Nominal	Yes/No
General health issues—allergies	Q10_2	Nominal	Yes/No
General health issues—cardiovascular	Q10_3	Nominal	Yes/No
General health issues—gastric	Q10_4	Nominal	Yes/No
General health issues—renal	Q10_5	Nominal	Yes/No
General health issues—arthritis	Q10_6	Nominal	Yes/No
General health issues—osteoporosis	Q10_7	Nominal	Yes/No
General health issues—lung diseases	Q10_8	Nominal	Yes/No
General health issues—depression	Q10_9	Nominal	Yes/No
General health issues—other	Q10_10	Nominal	string
Normal blood sugar	Q11	Nominal	Yes/No/I don’t know
Normal cholesterol	Q12	Nominal	Yes/No/I don’t know
Use of medication	Q13_1	Nominal	Yes/No
Use of medication—what medication	Q13_2	Nominal	string
Frequency of dentist visits	Q14	Ordinal	1 to 4
Dental plaque present	Q15	Nominal	Yes/No
Plaque coloring	Q16	Nominal	Yes/No
Dental mobility	Q17_0	Nominal	Yes/No
Dental mobility—absent	Q17_1	Nominal	Yes/No
Dental mobility—medium	Q17_2	Nominal	Yes/No
Dental mobility—advanced	Q17_3	Nominal	Yes/No
Dental mobility—localized	Q17_4	Nominal	Yes/No
Dental mobility—general	Q17_5	Nominal	Yes/No
Age of diagnosis for dental mobility	Q18	Scale	numeric
Gum diseases in family members	Q19	Nominal	Yes/No/I don’t know
Age of diagnosis for paradental disease	Q20	Ordinal	0 to 5
Frequency of gum bleeds	Q21	Ordinal	1 to 3
Dental retractions	Q22	Nominal	Yes/No
Cause of dental retractions	Q23	Nominal	4 categories
Orthodontic treatment	Q24	Nominal	Yes/No
Dental hygiene—dental floss	Q25_1	Nominal	Yes/No
Dental hygiene—manual brushing	Q25_2	Nominal	Yes/No
Dental hygiene—mechanical brushing	Q25_3	Nominal	Yes/No
Dental hygiene—Superfloss	Q25_4	Nominal	Yes/No
Dental hygiene—interdental brushing	Q25_5	Nominal	Yes/No
Dental hygiene—oral douch	Q25_6	Nominal	Yes/No
Dental hygiene—-other	Q25_7	Nominal	string
Time spent on hygiene	Q26	Ordinal	1 to 6
Frequency of dental floss	Q27	Ordinal	1 to 5
Frequency of mouthwash (last 7 days)	Q28	Ordinal	0 to 4
Frequency of other methods of hygiene (last 7 days)	Q29	Ordinal	0 to 4
Think you have gum problems	Q30	Nominal	Yes/No/I don’t know
Gingivitis treatment	Q31	Nominal	Yes/No/I don’t know
Eating habits	Q32	Nominal	4 categories
Where do you eat	Q33	Nominal	3 categories
Eat regularly	Q34	Nominal	Yes/No
Daily menu composition	Q35	Nominal	2 categories
Amount of water per day	Q36	Ordinal	1 to 5
Preferred diet	Q37	Nominal	2 categories
Are you stressed	Q38_1	Nominal	Yes/No/I don’t know
Stress sources—family	Q38_2	Nominal	Yes/No
Stress sources—work	Q38_3	Nominal	Yes/No
Stress sources—don’t know	Q38_4	Nominal	Yes/No
Stress sources—other	Q38_5	Nominal	Yes/No
Dental extractions	Q39	Ordinal	1 to 5
Happy with how teeth look	Q40	Ordinal	1 to 5
Bad breath	Q41	Nominal	Yes/No
Bad breath—noticed by other	Q42	Nominal	Yes/No/I don’t know
Chewing difficulties (last 6 months)	Q43_1	Ordinal	1 to 5
Speech difficulties (last 6 months)	Q43_2	Ordinal	1 to 5
Cleaning difficulties (last 6 months)	Q43_3	Ordinal	1 to 5
Sleep difficulties (last 6 months)	Q43_4	Ordinal	1 to 5
Smiling difficulties (last 6 months)	Q43_5	Ordinal	1 to 5
Weight	Q44_1	Scale	numeric
Height	Q44_2	Scale	numeric
Frequency of drinking alcohol	Q45	Ordinal	1 to 5
How many standard drinks per day	Q46	Ordinal	1 to 5
How often you drink 6 standard drinks once	Q47	Ordinal	1 to 5
Lost interest in common activities	Q48	Nominal	Yes/No
Feeling of sadness	Q49	Nominal	Yes/No
Do you practice sports?	Q50	Nominal	Yes/No
Pronunciation difficulties (last 7 days)	Q51_1	Ordinal	1 to 5
Taste difficulties (last 7 days)	Q51_2	Ordinal	1 to 5
Life dissatisfaction (last 7 days)	Q51_3	Ordinal	1 to 5
Relaxation difficulties (last 7 days)	Q51_4	Ordinal	1 to 5
More tension due to problems in last 7 days	Q51_5	Ordinal	1 to 5
Interrupt meals due to problems in last 7 days	Q51_6	Ordinal	1 to 5
Unpleasant when eating due to problems in last 7 days	Q51_7	Ordinal	1 to 5
Annoyance due to problems in last 7 days	Q51_8	Ordinal	1 to 5
Difficulty in fulfilling daily tasks due to problems in last 7 days	Q51_9	Ordinal	1 to 5
Powerless due to problems in last 7 days	Q51_10	Ordinal	1 to 5
Shame due to problems in last 7 days	Q51_11	Ordinal	1 to 5
Unsatisfactory diet due to problems in last 7 days	Q51_12	Ordinal	1 to 5
Pain in the mouth area in the last 7 days	Q51_13	Ordinal	1 to 5
Feeling unsafe regarding teeth	Q51_14	Ordinal	1 to 5
Are you satisfied with the health of your teeth?	Q52	Ordinal	1 to 5
Detachable prosthetics	Q53	Nominal	3 categories
Do dental problems affect your life quality?	Q54_1	Nominal	Yes/No
Do dental problems affect your life quality?—How much?	Q54_2	Ordinal	1 to 5
Mouth dryness	Q55	Nominal	Yes/No
Bad habits	Q56	Nominal	6 categories
Feeling of bad breath	Q57	Nominal	Yes/No
Bad breath—noticed by other	Q58	Nominal	Yes/No
Minimum amount to pay for an app	Q59	Ordinal	1 to 6
Usefulness of an app	Q60	Ordinal	1 to 5
Maximum amount to pay for an app	Q61	Ordinal	1 to 6
